# Non-structural protein-1 is required for West Nile virus replication complex formation and viral RNA synthesis

**DOI:** 10.1186/1743-422X-10-339

**Published:** 2013-11-18

**Authors:** Soonjeon Youn, Rebecca L Ambrose, Jason M Mackenzie, Michael S Diamond

**Affiliations:** 1 Department of Medicine, Washington University School of Medicine, Saint Louis, MO 63110, USA; 2 Department of Molecular Microbiology, Washington University School of Medicine, Saint Louis, MO 63110, USA; 3 Department of Pathology and Immunology, Washington University School of Medicine, Saint Louis, MO 63110, USA; 4 Department of Microbiology and Immunology, University of Melbourne, Parkville, Melbourne 3010, Australia

**Keywords:** Flavivirus, Replication, Infection, Trans-complementation

## Abstract

**Background:**

Flavivirus NS1 is a non-structural glycoprotein that is expressed on the cell surface and secreted into the extracellular space, where it acts as an antagonist of complement pathway activation. Despite its transit through the secretory pathway and intracellular localization in the lumen of the endoplasmic reticulum and Golgi vesicles, NS1 is as an essential gene for flavivirus replication. How NS1 modulates infection remains uncertain given that the viral RNA replication complex localizes to the cytosolic face of the endoplasmic reticulum.

**Methods and Results:**

Using a trans-complementation assay, we show that viruses deleted for NS1 (∆-NS1) can be rescued by transgenic expression of NS1 from West Nile virus (WNV) or heterologous flaviviruses in the absence of adaptive mutations. In viral lifecycle experiments, we demonstrate that WNV NS1 was not required for virus attachment or input strand translation of the infectious viral RNA, but was necessary for negative and positive strand RNA synthesis and formation of the endoplasmic reticulum-associated replication complex.

**Conclusions:**

WNV RNA lacking intact NS1 genes was efficiently translated but failed to form canonical replication complexes at early times after infection, which resulted in an inability to replicate viral RNA. These results expand on prior studies with yellow fever and Kunjin viruses to show that flavivirus NS1 has an essential co-factor role in regulating replication complex formation and viral RNA synthesis.

## Background

Members of the *Flavivirus* genus are the most important arthropod-borne viruses causing disease in humans. This genus includes viruses (West Nile (WNV), Japanese encephalitis (JEV), yellow fever (YFV), and dengue (DENV) viruses) that are endemic in several parts of the world [1]. Flavivirus infection causes severe disease in humans including hemorrhagic fever, shock syndrome, liver failure, flaccid paralysis, and encephalitis. The ~10.7 kilobase single-stranded positive sense flavivirus RNA genome is translated as a single polyprotein, which is cleaved into three structural proteins (C, prM/M, E) and seven nonstructural (NS) proteins (NS1, NS2A, NS2B, NS3, NS4A, NS4B, NS5) by virus- and host-encoded proteases. Flavivirus RNA replication occurs along the cytosolic face of the endoplasmic reticulum (ER) and requires the enzymatic actions of several NS proteins including the viral helicase and protease (NS3) and RNA-dependent RNA polymerase (NS5).

Flavivirus NS1 is a multi-functional 48 kDa non-structural glycoprotein [2] that is synthesized as a monomer, dimerizes after post-translational modification in the lumen of the ER, and accumulates in extracellular fluid as a hexamer with a lipid core [3-7]. Flaviviruses in the JEV serocomplex also express NS1′, an additional form of NS1 with a 52 amino acid C-terminal extension, which is the result of ribosomal frame shift due to a conserved pseudoknot in the 5' end of the NS2A gene [8,9]. Although its precise function remains unknown, the specific deletion of NS1′ results in attenuation of neurovirulence of both WNV and JEV [9,10]. NS1 is expressed on the surface of cells through at least two mechanisms: (a) soluble NS1 binds back to the plasma membrane of uninfected and infected cells [11] through interactions with sulfated glycosaminoglycans [12]; and (b) NS1 also is expressed directly on the plasma membrane of infected cells although it lacks a canonical transmembrane domain or targeting motif for cellular membranes. The mechanism of direct cell surface expression remains uncertain although some fraction may be linked through an atypical glycosyl-phosphatidylinositol anchor [13,14] or lipid rafts [15]. NS1 has immune evasive functions in the extracellular space, on the surface of cells, and within cells, as it binds to complement proteins and regulators and antagonizes their functions [16-18] and possibly, disrupts TLR3 signaling pathways [19].

Despite its transit through the secretory pathway, NS1 is an essential gene and modulates early viral RNA replication [20-22]. Deletion of NS1 from the viral genome abrogates replication, although an NS1-deleted virus (∆NS1) can be complemented *in trans* by ectopic expression of NS1. Prior studies have suggested that the essential intracellular function of NS1 is due to its ability to regulate negative strand synthesis of viral RNA [22]. Genetic and biochemical studies have suggested that NS1 interacts with NS4A and NS4B, transmembrane viral proteins that span the ER, which could integrate key signals from NS1 for RNA replication occurring in the cytoplasm [23,24].

Here, we explored the function of intracellular NS1 in regulating flavivirus replication. We confirmed prior studies [22,23,25] showing that flaviviruses containing an in-frame deletion in NS1 fail to replicate efficiently in cells. In contrast to earlier studies, deletion viruses were rescued by transgenic expression of homologous (WNV) or heterologous (YFV, DENV, JEV, or Saint Louis encephalitis virus (SLEV)) NS1, the latter occurring in the absence of adaptive mutations. Intracellular NS1 played a key role in regulating RNA synthesis and replication complex formation. Viral RNA lacking intact NS1 genes were efficiently translated but failed to form canonical replication complexes at early times after infection, which resulted in an inability to accumulate negative strand viral RNA intermediates.

## Results

### WNV lacking NS1 expression is replication-incompetent but can be trans-complemented

**Figure 1:**
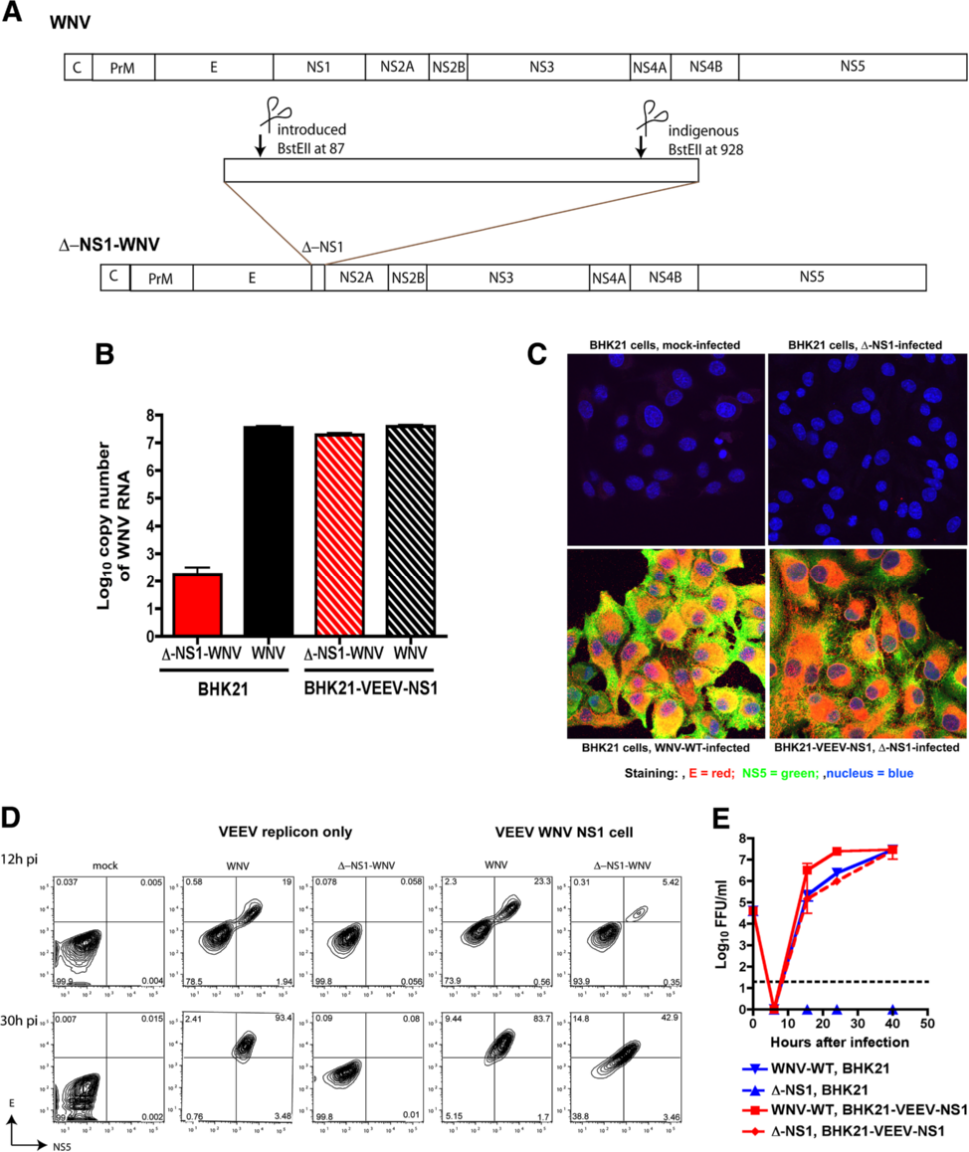
**Trans-complementation of ∆-NS1-WNV with ectopically expressed WNV NS1. A.** Scheme for construction of ∆-NS1-WNV. Nucleotides 87 to 928 of the NS1 gene were deleted after restriction digest with the BstEII enzyme. **B.** Recovery of WNV RNA in supernatant after transfection of ∆-NS1-WNV or WNV-WT RNA into BHK21 or BHK21-VEEV-WNV NS1 cells. 12 hours post transfection, supernatant was harvested and assessed for levels of viral RNA by qRT-PCR of the E gene. The results are the average of two independent experiments performed in triplicate. **C-D.** Immunofluorescence **(C)** or flow cytometry **(D)** staining of WNV E and NS5 antigen after mock infection or infection of ∆-NS1-WNV or WNV-WT in BHK21 or BHK21-VEEV-WNV NS1 cells. Cells were infected at an MOI of 5, and at 26 hr post infection, cells were fixed, permeabilized, and stained with anti-E (red), anti-NS5 (green), or a nuclear stain (blue). Results are representative of several independent experiments. **E.** Single-step growth kinetics of ∆-NS1-WNV or WNV-WT in BHK21 or BHK21-VEEV-WNV NS1 cells. Cells were infected at an MOI of 5 and supernatants were titrated on BHK21-VEEV-WNV NS1 cells by focus-forming assay. The dashed line indicates the limit of detection of the assay. Results are the average of several independent experiments performed in triplicate. Error bards indicate standard deviations.

Prior studies showed that deletion of NS1 from infectious cDNA clones of Kunjin (KUNV) [25] or yellow fever (YFV) [22] flaviviruses impaired virus replication, which suggested that NS1 was an essential gene for infection. These studies also demonstrated that KUNV and YFV lacking NS1 could be complemented *in trans* by ectopic expression of the homologous NS1. To assess the role of NS1 in infection of a North American strain of WNV (WNV-New York 1999), we generated an infectious cDNA clone (∆-NS1-WNV) that deleted 840 nucleotides in-frame (from 87 to 928) of the NS1 gene (Figure 1A and see Methods); this left a small fragment of NS1 consisting of the 86 N-terminal and 201 C-terminal nucleotides.

After *in vitro* transcription and electroporation of wild type (WNV-WT) or ∆-NS1-WNV RNA into BHK21 or BHK21 VEEV-WNV-NS1 cells (the latter express WNV NS1 *in trans* from an autonomously propagating VEEV replicon [24]), supernatants were harvested and evaluated for viral RNA by quantitative RT-PCR (qRT-PCR). WNV-WT RNA was recovered readily from supernatants of both BHK21 or BHK21 VEEV-WNV-NS1 cells. In comparison, while similar amounts of ∆-NS1-WNV RNA were present in the supernatant of BHK21 VEEV-WNV-NS1 cells, much lower levels (>10^5^-fold) were recovered from the supernatants of BHK21 cells (Figure 1B). To confirm that ∆-NS1-WNV could be rescued only in cells expressing NS1, supernatant from BHK21 VEEV-WNV-NS1 cells transfected with ∆-NS1-WNV was used to infect a second set of BHK21 or BHK21 VEEV-WNV-NS1 cells. At 30 hours after infection, cells were analyzed for WNV E and NS5 protein expression by confocal microscopy and flow cytometry. Only BHK21 VEEV-WNV-NS1 but not BHK21 cells infected with ∆-NS1-WNV showed evidence of productive infection and WNV antigen expression (Figure 1C and D).

To address the relative efficiency of trans-complementation of ∆-NS1-WNV, growth kinetics of infectious WNV-WT and ∆-NS1-WNV were compared on BHK21 and BHK21 VEEV-WNV-NS1 cells by focus-forming assay (Figure 1E). Consistent with the immunofluorescence data, ∆-NS1-WNV failed to produce infectious foci on BHK21 cells confirming that it fails to replicate efficiently in the absence of NS1 expression. However, ∆-NS1-WNV growth kinetics on BHK21 VEEV-WNV-NS1 cells were comparable to WNV-WT infection on BHK21 cells, with no appreciable difference observed at any time point examined (*P* > 0.5). WNV-WT also replicated more efficiently on BHK21 VEEV-WNV-NS1 cells (10 to 15-fold higher levels compared to BHK21 cells, *P* < 0.01) at early time points (16 and 24 hours) after infection, suggesting a difference in infectivity of the cells was due to expression of the VEEV replicon or ectopic expression of WNV-NS1. Experiments were repeated with cells expressing a VEEV replicon that lacked NS1 (BHK21-VEEV empty). As these cells sustained similar levels of WNV-WT infection compared to the parent BHK21 cells (data not shown), ectopic expression of NS1 improves infectivity of WNV-WT, at least during the early stages of replication.

### NS1 protein expression is required for trans-complement of ∆-NS1-WNV

**Figure 2:**
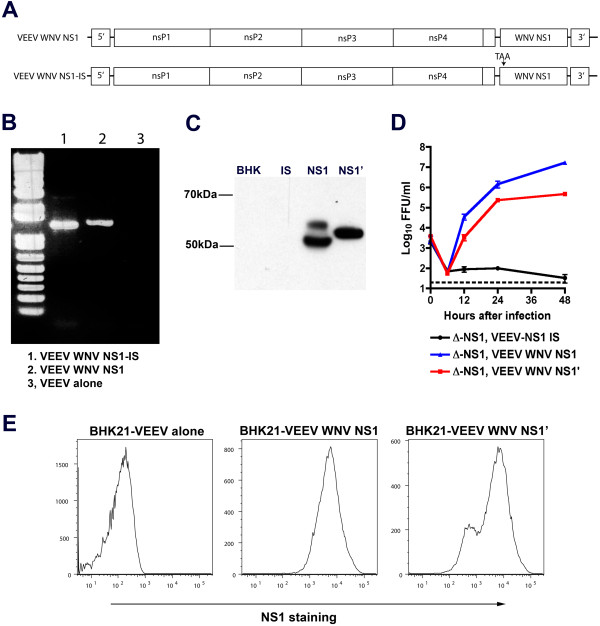
**Trans-complementation of ∆-NS1-WNV with non-translated NS1 or ectopically expressed WNV NS1′. A.** Scheme for construction of a VEEV replicon that encodes a non-translated form of NS1 containing an internal stop codon at the 5′ end. **B.** Agarose gel electrophoresis showing PCR of the cDNA of full-length NS1 and NS1 with an internal stop codon. The bands are slightly different in size due to the use of distinct primer sets. **C.** Western blot of NS1 in BHK21 cells propagating replicons encoding NS1-IS (internal stop), NS1, or NS1′. NS1 migrates as a doublet reflecting intracellular NS1 (non-glycosylated, lower band) and cell-surface NS1 (glycosylated, upper band). NS1′ in cell lysates reflects only an intracellular non-glycosylated form, as it is not expressed appreciably on the cell surface (data not shown). **D.** Growth kinetics of ∆-NS1-WNV in BHK21-VEEV-WNV NS1, BHK21-VEEV-WNV-NS1′, or BHK21-VEEV WNV NS1 IS cells. Cells were infected at an MOI of 1, and supernatants were titrated on BHK21-VEEV-WNV NS1 cells by focus-forming assay. The dashed line indicates the limit of detection of the assay. Results are the average of two independent experiments performed in triplicate. Error bards indicate standard deviations. **E.** Expression of NS1 and NS1′ in the VEEV replicon in BHK21 cells, as judged by (*left*) flow cytometry. Experiments in panels **B, C, and E** reflect one of several independent experiments.

Although flaviviruses lacking NS1 can be complemented *in trans* when NS1 is introduced as part of plasmid or autonomously propagating replicon, it remains unclear whether this is due to expression of NS1 protein or the presence of RNA elements in the NS1 gene. Indeed, RNA elements in the flavivirus genome can bind host factors that are required for replication [26,27]. To evaluate this hypothesis, a stop codon was inserted into the codon of the eighth amino acid of NS1 after the signal peptide and cloned into the VEEV replicon (Figure 2A). BHK21 cells propagating the VEEV-WNV-NS1-internal stop (IS) replicon transcribed NS1 RNA (Figure 2B) but did not express protein (Figure 2C): flow cytometric analysis showed that NS1 protein was produced in BHK21 VEEV-WNV-NS1 but not BHK21 VEEV-WNV-NS1-IS cells (data not shown). When BHK21 VEEV-WNV-NS1-IS cells were infected with ∆-NS1-WNV, infectious virus was not recovered from the supernatant (Figure 2D). Thus, the presence of an NS1 RNA transcript was not sufficient to trans-complement the infectivity defect associated with a deletion of NS1. Rather, NS1 protein is required for WNV replication.

### NS1′ trans-complements ∆-NS1 viruses

The JEV serocomplex of flaviviruses produces a second NS1 species, NS1′, which is generated as a product of a ribosomal frameshift [9,10,28]. As NS1′ reportedly contributes to the pathogenesis of WNV [9], we tested whether its expression would complement the infectivity of ∆-NS1. We ectopically expressed NS1′ (amino acids 768 to 1195 of the viral coding sequence) in a VEEV replicon in BHK21 cells (Figure 2C). Intracellular expression levels of NS1′ were comparable although slightly lower than that achieved in cells ectopically expressing NS1 (Figure 2E). Viral growth analysis revealed that transgenic expression of NS1′ also could complement the replication defect associated with ∆-NS1-WNV infection (Figure 2D).

### A deletion of WNV NS1 is complemented by NS1 from different flaviviruses

**Figure 3:**
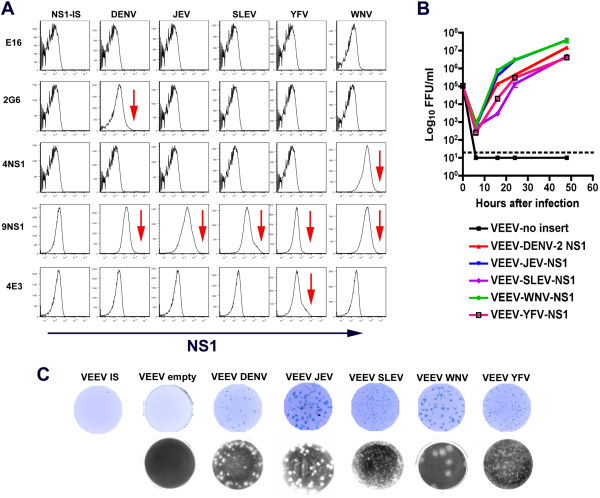
**Trans-complementation of **∆**-NS1-WNV by flavivirus NS1. A.** Expression of homologous or heterologous flavivirus NS1 in BHK21 cells using VEEV replicons. WNV (wild type or internal stop (IS), DENV-2, JEV, SLEV, or YFV NS1 were cloned and expressed in VEEV replicons and stable cells propagating these were generated. Cells were stained for NS1 expression with an irrelevant control MAb (E16, anti-WNV E), an anti-DENV-2 NS1 MAb (2G6), anti-WNV NS1 MAb (4NS1), a cross-reactive NS1 MAb (9NS1), and anti-YFV NS1 MAb (4E3). The results are representative of several independent experiments. Red arrows indicate positive expression that was over levels of the negative control MAb. **B.** Growth kinetics of ∆-NS1-WNV in BHK21-VEEV cells expressing WNV, DENV-2, SLEV, JEV, YFV NS1, or no insert (empty). Cells were infected at an MOI of 1, and supernatants were titrated on BHK VEEV WNV NS1 cells by focus-forming assay. The dashed line indicates the limit of detection of the assay. Results are the average of two independent experiments performed in triplicate. Error bards indicate standard deviations (most of which are smaller than the indicated symbols). **C.** Examples of (*top*) focus-forming or (*bottom*) plaque assays with ∆-NS1-WNV trans-complemented in BHK21-VEEV cells expressing WNV, DENV-2, SLEV, JEV, or YFV NS1. One representative experiment of three is shown.

A prior study showed that YFV with a deletion in NS1 was not trans-complemented by DENV-2 NS1 without a compensatory adaptive mutation in the NS4A gene [23]. To test whether ∆-NS1-WNV could be complemented by NS1 from other flaviviruses, we expressed JEV, SLEV, DENV-2, and YFV NS1 using distinct VEEV replicons. Expression of each flavivirus NS1 was confirmed by flow cytometry using species-specific or cross-reactive (e.g., 9NS1) anti-flavivirus NS1 MAbs (Figure 3A). ∆-NS1-WNV that was prepared in BHK21 VEEV-WNV-NS1 cells was titered by plaque and focus-forming assays directly on BHK21 cells expressing JEV, SLEV, DENV-2, or YFV NS1; this approach limits the development of adaptive mutations. Each heterologous flavivirus NS1 expressed from VEEV replicons trans-complemented ∆-NS1-WNV (Figure 3B). However, DENV-2, YFV, and SLEV NS1 trans-complemented ∆-NS1-WNV with smaller plaques size or lower number (5 to 240-fold) of infectious foci compared to cells expressing WNV NS1 (Figure 3C); these differences in relative infectivity could not be attributed to lower expression of heterologous NS1 (Figure 3A). Only cells expressing JEV NS1, which is the most closely related to WNV (~78% identity at the amino acid level), supported the large plaques and high viral yield that were seen with the complementing cell expressing WNV NS1. Given that adaptive mutations were required for trans-complementation of ∆-NS1 YFV with NS1 from heterologous flaviviruses [23], we sequenced the complete genomes of ∆-NS1 secreted WNV from cells expressing WNV, DENV-2, or YFV NS1. No specific mutation was identified in viruses produced by trans-complementing cells expressing DENV-2 or YFV NS1 compared to WNV NS1 (data not shown). Thus, heterologous flavivirus NS1 can trans-complement ∆-NS1 WNV without further passage or adaptation.

### ∆-NS1-WNV can enter cells and undergo input strand translation

**Figure 4:**
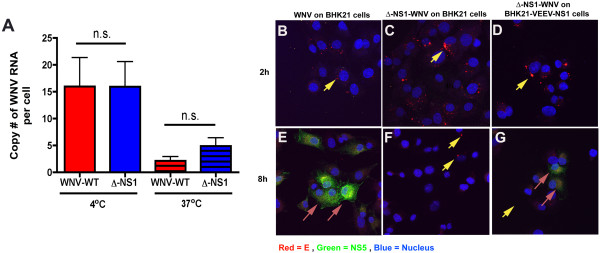
**∆-NS1-WNV virus does not have an attachment defect. A.** Direct binding to BHK21 cells. An MOI of 1 of WNV-WT or ∆-NS1-WNV was incubated at 4°C or 37°C with BHK21 cells for one hour. Unbound virus was removed by centrifugation and washing, and bound and/or internalized virus was quantified after cell lysis and RNA purification using qRT-PCR and a primer and probe set specific for positive strand specific WNV RNA. Values were normalized to 18S rRNA levels to account for possible differences in cell number. Results are the average of three independent experiments, and differences were not statistically significant. **B-G.** BHK21 **(B-C and E-F)** or BHK21 VEEV NS1 **(D, G)** were infected with WNV-WT **(B,E)** or ∆-NS1-WNV **(C-D, and F-G)** at an MOI of 5 for one hour and then unattached virus was removed by extensive washing. Two (*top panels*) or eight (*bottom panels*) hours later, cells were fixed, permeabilized and stained with anti-E (red) or anti-NS5 (green) antibodies, or a nuclear stain (blue). Yellow arrows indicate E protein in a punctate staining pattern prior to replication, consistent with virus that is entering cells through an endocytic pathway. Magenta arrows denote E and NS5 staining that occurs after viral replication has ensued. The results are representative of several independent experiments.

To define the stage in the viral lifecycle that was affected by a deletion in NS1 detailed cellular experiments were undertaken. Initially, we assessed whether trans-complemented ∆-NS1-WNV efficiently attached and entered cells, BHK21 cells were incubated with the same MOI of WNV or ∆-NS1-WNV for one hour at 4°C or 37°C. After extensive washing, bound virus was quantified by measuring genome copy number by qRT-PCR against the positive strand of WNV RNA. At the same MOI, ∆-NS1-WNV showed equivalent levels of attachment to or entry of cells compared to WNV-WT (Figure 4A).

We next assessed steps prior to viral RNA synthesis and replication complex formation by evaluating the amount of viral antigen present in the cell at times that preceded RNA replication. At the same MOI of 5, WNV or ∆-NS1-WNV was added to BHK21 or BHK21 VEE-WNV-NS1 cells. Virus was incubated for one hour at 37°C, and unbound virus was removed after extensive washing. Virus internalization was tracked by confocal microscopy using an anti-WNV MAb against the E protein. At two hours after WNV and ∆-NS1-WNV infection, punctate staining of E protein from was observed in BHK21 and BHK21 VEE-WNV-NS1 cells (Figure 4B-D), consistent with flavivirus entry through endosomal pathways [29,30]. By 8 hours, BHK21 cells infected with WNV-WT or BHK21 VEE-WNV-NS1 cells infected with WNV-WT or ∆-NS1-WNV showed evidence of nascent E and NS5 protein synthesis, with a reticular pattern of staining consistent with its initial localization to the ER (Figure 4E-G, and data not shown). In contrast, at 8 hours, BHK21 cells infected with ∆-NS1-WNV showed a loss of E protein staining and no NS5 staining, as would be expected with a failure to replicate positive strand viral RNA.

**Figure 5:**
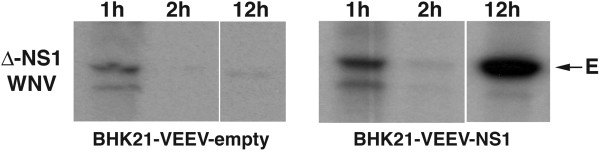
**Input strand translation of ∆-NS1-WNV in BHK21-VEEV-empty and BHK21-VEEV-WNV-NS1 cells.** An MOI of 5 of ∆-NS1-WNV was incubated at 37°C with (*left*) BHK21-VEEV-empty or (*right*) BHK21-WNV-VEEV-NS1 cells. At 1, 2, or 12 hours, cells were starved in cysteine-methionine deficient medium for 30 minutes, and then pulse-labeled with ^35^S-cysteine-methionine for an additional 30 minutes. Cells were lysed (see Methods), immunoprecipitation was performed with an anti-E protein MAb, proteins were electrophoresed, and gels were subjected to autoradiography. The arrow indicates the mobility of the E protein band. The results are representative of three independent experiments.

To further define defects in the viral lifecycle associated with a deletion of NS1, we assessed input strand translation. To differentiate input strand protein during the initial translation period, cells were labeled biosynthetically and WNV E protein was immunoprecipitated from lysates at specified times. BHK21-VEEV-NS1 cells or BHK21-VEEV-empty cells were infected with ∆-NS1-WNV. One hour later, cells were placed in cysteine-methionine-free medium, pulsed for 30 minutes with ^35^S-methionine-cysteine, and nascently generated E protein was detected by immunoprecipitation (Figure 5). As E protein translation was observed in ∆-NS1-WNV-infected BHK21-VEEV-empty and BHK21-VEEV-NS1 cells, NS1 was not required in *cis* for initial input strand translation. By two hours, however, translation of the input strand was no longer readily detected likely due to recruitment of the viral RNA into the replication complex. By later time points (e.g., 12 hours), E protein translation again was observed in ∆-NS1-WNV-infected BHK21-VEEV-NS1 cells but not in cells lacking transgenic expression of NS1.

### ∆-NS1-WNV shows a defect in viral RNA synthesis

**Figure 6:**
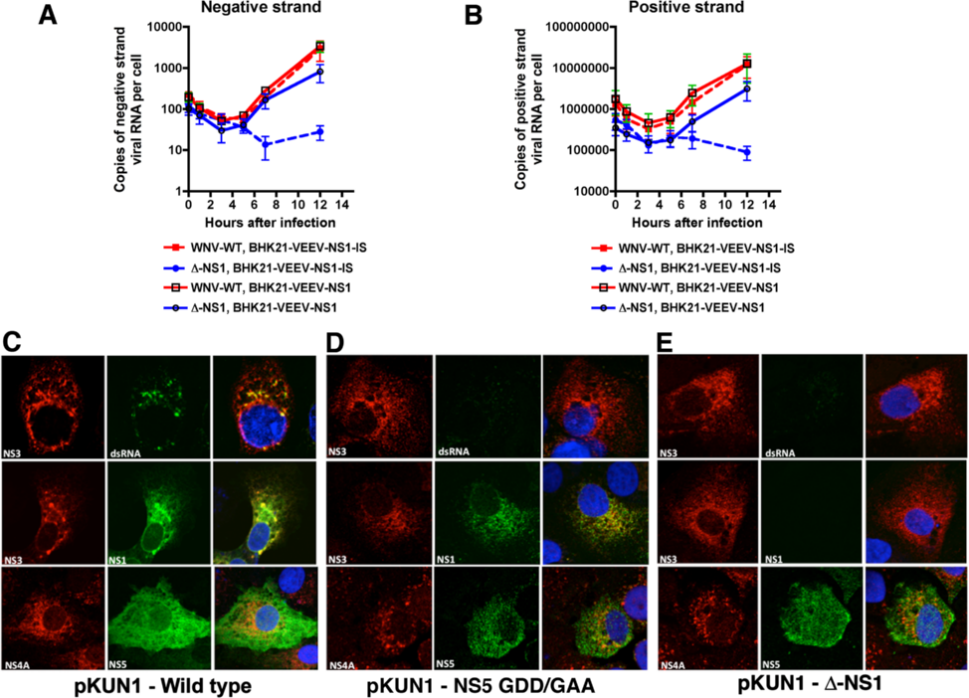
**A deletion of WNV NS1 affects viral RNA synthesis and formation of the replication complex. A-B.** Measurement of negative **(A)** or positive **(B)** strand viral RNA in BHK21-VEEV-NS1 or BHK21-VEEV-NS1-IS (premature stop codon) cells infected with WNV-WT or ∆-NS1-WNV. The indicated cells were infected with WNV-WT or ∆-NS1-WNV at an MOI of 5. At the indicated time points, viral RNA was harvested and strand-specific qRT-PCR [31,32] was performed. The results are the average of two independent performed in triplicate. The very low levels of negative strand viral RNA at input (t = 0) have been reported previously for DENV and WNV [33,34], and possibly reflect delivery of viral RNA in exosomes or defective viruses with inappropriate packing of RNA intermediates. **C-E.** Vero cells were transfected with CMV-launched infectious cDNA clones of **(C)** wild type KUNV (pKUN1-wild type), **(D)** KUNV with a mutation in NS5 that abolishes RNA polymerase activity (pKUN1-NS5 GDD/GAA), or **(E)** ∆-NS1-WNV KUNV (pKUN1-∆-NS1). 48 hours later, cells were co-stained with antibodies against *(top panels*) NS3 and dsRNA, (*middle panels*) NS3 and NS1, or (*bottom panels*) NS4A and NS5. Nuclei were stained blue with DAPI. Images were processed by confocal microscopy. Representative images from three independent experiments are shown.

NS1 is believed to be required for flavivirus infection because of an essential function in viral RNA translation or replication [22]. To address whether ∆-NS1-WNV had a defect at or before RNA synthesis, we monitored viral RNA accumulation over time using asymmetric strand-specific qRT-PCR [31] (Figure 6A and B). Notably, WNV-WT and ∆-NS1-WNV showed a similar trend in BHK21 VEEV-WNV-NS1 cells through 5 hours after infection with decay in the levels of negative and positive strand RNA. However, by 7 hours after WNV-WT or ∆-NS1-WNV infection, negative and positive strand RNA increased in BHK21 VEE-WNV-NS1 cells, although the levels of viral RNA in ∆-NS1-WNV-infected cells did not reach those seen with WNV-WT. In comparison, in BHK21 VEE-WNV-NS1-IS (internal stop) cells, WNV-WT showed increased negative and positive strand RNA whereas ∆-NS1-WNV did not.

### ∆-NS1-WNV does not form a replication complex

**Figure 7:**
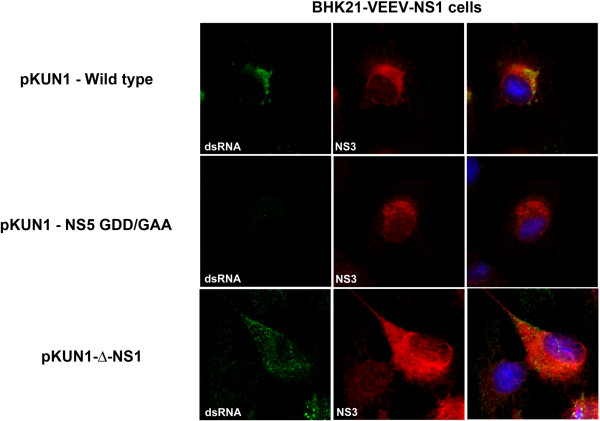
**Transgenic expression of NS1 rescues the ability of an NS1 deletion virus to form dsRNA replication complexes.** BHK21-VEEV-NS1 cells were transfected with CMV-launched infectious cDNA clones of pKUN1-WT, pKUN1-NS5 GDD/GAA, or pKUN1-∆-NS1. 48 hours later, cells were co-stained with antibodies against dsRNA *(left panels*) or NS3 (*middle panels*). Nuclei were stained blue with DAPI and images were merged (*right panels*). Images were processed by confocal microscopy. Representative images from three independent experiments are shown.

Given the absence of viral RNA accumulation in cells infected with ∆-NS1-WNV, we hypothesized that NS1 might be essential for formation of the viral replication complex that forms on the ER membrane. To begin to assess this, we took advantage of an existing CMV launched infectious cDNA clone of the Kunjin strain of WNV (pKUN1) [35], and generated ∆-NS1 (deletion of amino acids 5 to 298 of NS1) and NS5 polymerase dead (GDD to GAA at amino acids 666 and 667) mutant plasmids [36]. We used this approach so viral proteins could accumulate in the cell in the absence of a requirement for active RNA replication. Two days after transfection, Vero cells were analyzed by immunofluorescence microscopy for co-localization of NS3 and NS5 in the replication complexes. Transfection of the parent cDNA clone (pKUN1) resulted in the co-localization of NS3, NS5, and dsRNA in puncta at the ER (Figure 6C). In contrast, transfection of Vero cells with pKUN1-NS5-GAA or pKUN1-∆-NS1 resulted in accumulation of NS3 and NS5 without recruitment of NS3 into puncta at the ER, the sites of the viral replication complex (Figure 6D and E). Moreover, labeling of double-stranded RNA was absent in pKUN1-NS5-GAA or pKUN1-∆-NS1 transfected cells. In comparison, transfection of BHK21-VEEV-NS1 complementing cells with pKUN1-∆-NS1 but not pKUN1-NS5-GAA resulted in the formation of replication complexes (identified by dsRNA) that co-localized with NS3 protein (Figure 7). Collectively, these data confirm that NS1 is required for viral RNA synthesis, and without this, replication complexes fail to form efficiently.

## Discussion

Flavivirus NS1 is a non-structural glycoprotein that is expressed on the plasma membrane of infected cells and secreted into the extracellular space. Secreted NS1 antagonizes complement function in solution and on the plasma membrane surface, and thus serves an immune evasion function [16-18,37]. Within the cell, even though it is localized to the lumen of the ER and viral replication occurs on the cytosolic face of the ER, NS1 is an essential gene that regulates early viral RNA replication [22,38]. How this occurs has been poorly understood, although prior studies suggested NS1 is required for a step proximal to viral RNA synthesis [22], possibly through interactions with the viral transmembrane proteins NS4A [23] or NS4B [24], both of which have direct proximity to the replication complex on the cytosolic face of the ER membrane. In the current study, using two WNV strains that lack a functional NS1 gene and a trans-complementation system, we show that while WNV NS1 was not required for input strand translation of the infectious viral RNA, it was necessary for negative and positive strand RNA synthesis and formation of the replication complex.

While the ability of ectopically-expressed NS1 to trans-complement flaviviruses (WNV or YFV) containing gene deletions in NS1 has been demonstrated previously [22,25], our studies show uniquely for WNV that heterologous NS1 from DENV, JEV, SLEV, and YFV all can serve this function. Each heterologous flavivirus NS1 expressed from VEEV replicons trans-complemented ∆-NS1-WNV without further passage. Nonetheless, we observed differences in efficiency, as trans-complementation with DENV, YFV, and SLEV NS1 resulted in smaller plaques sizes and/or lower numbers of infectious foci compared to cells ectopically expressing NS1 from homologous (WNV) or the most closely related (JEV) flaviviruses. In comparison, a prior study showed that ∆-NS1 YFV could not be trans-complemented by DENV-2 NS1 without adaptation, possibly due to an inability to interact with NS4A and the remainder of the YFV replication complex [23]. Thus, the requirements for regulating replication by NS1 appear conserved enough to allow for trans-complementation of ∆-NS1-WNV by NS1 from other mosquito-transmitted flaviviruses.

Our detailed kinetic experiments demonstrating that an absence of WNV NS1 affects viral RNA synthesis as judged by strand-specific qRT-PCR confirm and extend earlier results with ∆-NS1 YFV that were obtained by Northern blotting [22]. We show that trans-complemented ∆-NS1 WNV bound to and entered cells, and the input strand of genomic RNA was translated at early time points. Moreover, by using a CMV launched wild type and mutant (∆-NS1 or NS5 polymerase dead) WNV-KUNV, we showed that NS1 is required for formation of the replication complex and recruitment of other non-structural proteins (e.g., NS3) to the vesicle packets [39] associated with ER membranes; the strength of this experimental approach is that it allows for continued transcription and translation of viral non-structural genes in the absence of a requirement for active replication. However, it remains unclear whether the absence of replication complex after transfection of ∆-NS1 WNV-KUNV was due to the lack of production of dsRNA or the failure to recruit key viral and possibly, host proteins to the specialized ER membranes, which serve as the site of flavivirus replication.

## Conclusions

Our experiments establish that WNV NS1 contributes to viral replication. Whereas soluble NS1 functions to antagonize innate immune responses through interactions with complement or pathogen recognition receptors [16,17,19,37], intracellular NS1 serves a discrete purpose during viral RNA synthesis, possibly to help form or stabilize the replication complex. Ongoing studies are planned to define the precise molecular details by which NS1 executes functions at different stages of the flavivirus virus lifecycle.

## Methods

### Cells and viruses

BHK21 cells were grown in complete Dulbecco’s modified Eagle’s medium (DMEM) (supplemented with 10% fetal bovine serum (FBS), penicillin, streptomycin, 10 mM HEPES pH 7.3, and non-essential amino acids) in a 5% humidified CO_2_ incubator at 37°C. BHK21 cells propagating VEEV replicons were maintained in complete DMEM supplemented with 5 μg/ml of puromycin. Infection experiments were performed with the New York 1999 strain (385–99) of WNV, which was derived from an infectious cDNA clone [40], or WNV-KUNV, as detailed below.

### Generation of cells propagating VEEV replicons expressing flavivirus NS1

Full length NS1 including the N-terminal signal sequences from WNV (strain New York 1999, amino acids 768 to 1143), DENV-2 (strain D2S10, amino acids 776 to 1127), YFV (strain 17D, amino acids 760 to 1136), SLEV (strain GHA6, amino acids 766 to 1141), and JEV (strain 14-14-2, amino acids 771 to 1146) were amplified from viral RNA or infectious clone cDNA by RT-PCR and PCR using specific primers (Table 1), and cloned into pSTBlue TA (EMD Millipore). To clone the NS1′ form of WNV, one additional nucleotide (T) was introduced at the site of the −1 frame shift by QuickChange mutagenesis using the following primers: forward: 5′-GATATGATTGACCCTTTTTCAGTTGGGCCTTCTG-3′, reverse: 5′-CAGAAGGCCCAACTGAAAAAGGGTCAATCATATC-3′, this resulted in the generation of an NS1′ with an additional 52 amino acids at the C-terminus. After sequence verification, all NS1 genes were subcloned into a modified pSC-B W/VEEV shuttle vector and then into a VEEV replicon (pTC83new/Pac [41]; gift of I. Frolov) using an XbaI site. VEEV replicon plasmids encoding NS1 were linearized with MluI and used as templates for *in vitro* transcription using an SP6 DNA-dependent RNA polymerase mMESSAGE mRNA transcription kit (Ambion) according to the manufacturer’s instruction. RNA transcripts were introduced into cells using a GenePulser Xcell electroporator (Bio-Rad) at 850 V, 25 μF, and infinite Ω, and cells expressing VEEV replicons were selected with puromycin (5 μg/ml) over one week. Flavivirus NS1 expression was confirmed by flow cytometry as described below.

**Table 1:** Primers for cloning NS1 from different flaviviruses

**Primers**	**Sequence (5′-3′)**
WNV NS1 forward	GTGGATGGGCGGCCGCACCATGGATAGGTCCATAGCTCTCACGTTT
WNV NS1 reverse	CATTGACTGCGGCCGCTAAGCATTCACTTGTGACTGCAC
DENV NS1 forward	GCGGCCGCACCATGGTCTCACTGTCTGTGACACTAG
DENV NS1 reverse	TCAAGCTGTGACCAAGGAGTTGAC
YFV NS1 forward	GCGGCCGCACCATGGACATGACAATGTCCATGAGCA
YFV NS1 reverse	TCAAGCTGTAACCCAGGAGCGCACCAG
JEV NS1 forward	GCGGCCGCACCATGGACCGATCAATTGCTTTGGCC
JEV NS1 reverse	TCAAGCATGAACCTGTGATCTGACG
SLEV NS1 forward	GCGGCCGCACCATGGACAGGAGCATCTCGCTGACTC
SLEV NS1 reverse	TCAAGCTGTCACGCGAGATTTCACAAG

### Construction and production of ∆-NS1 WNV-NY

Using the pSTBlue vector containing WNV NS1 as a template, a second BstEII restriction enzyme site was introduced at nucleotide 87 of the WNV NS1 gene (from CATAGAC to GGTCACC) by site directed mutagenesis using the following primers: forward: 5′- GACACTGGGTGTGC**GGT**C**ACC**ATCAGCCGGCTCTAG-3′, reverse: 5′-CTAGAGCCGGCTGAT**GGT**G**ACC**GCACACCCAGTGTC-3′. Subsequently, after BstEII enzyme digestion and self-ligation, the 840 nucleotides of the NS1 gene (87 to 928) were removed although the reading frame of the NS1 deletion fragment (∆-NS1) remained intact. This ∆-NS1 fragment was subcloned into the two-plasmid system infectious cDNA clone of WNV-NY [40] to generate p∆-NS1-WNV. Using T7 DNA-dependent RNA polymerase, p∆-NS1-WNV was transcribed *in vitro* and transfected into BHK21 or BHK21 VEEV-WNV-NS1 cells. Flow cytometric studies with ∆-NS1 failed to show production of any residual truncated protein using anti-NS1 MAbs that recognize different regions of the intact protein.

### Trans-complementation plaque and focus-forming assays

The ability of different flavivirus NS1 to trans-complement ∆NS1-WNV was determined by serially diluting ∆-NS1-WNV (produced in cells ectopically expressing WNV NS1) and then infecting BHK21 cells propagating VEEV replicons expressing WNV, JEV, SLEV, DENV-2, or YFV NS1 for one hour at 37°C. Subsequently, DMEM containing final 4% FBS and 1% low melting agarose was overlaid and incubated an additional 3 days for plaque forming assay or DMEM containing final 4% FBS and 1% methylcellulose was overlaid and incubated for one day for the focus-forming assay. Plaques were stained with 1% crystal violet after fixation with 10% formaldehyde and then counted as described previously [42]. For the focus-forming assay, cells were fixed with 1% paraformaldehyde for 20 minutes. After removal of the methylcellulose, cells were washed three times with PBS, permeabilized with PBS supplemented with 1% saponin (w/v) and 1% BSA (w/v), and incubated with mouse E16 anti-WNV MAb [43] for three hours at room temperature or overnight at 4°C. Following three washes with PBS, cells were incubated for one hour with horseradish peroxidase-conjugated goat anti-mouse IgG (Sigma, 1/5,000 dilution), and after additional washes, foci were visualized with TrueBlue peroxidase substrate (KPL) and counted using a BioSpot Analyzer (C.T.L).

### Western blotting

Supernatants from BHK21 VEEV-NS1-IS, BHK21 VEEV-WNV-NS1, or BHK21 VEEV NS1′ cells were separated by 12% PAGE, transferred to nitrocellulose membrane using an iBlot dry blotting system (Invitrogen). Membranes were blocked for one hour at room temperature in 50 mM Tris, 150 mM NaCl, 0.05% Tween 20 (TBST) supplemented with 5% non-fat dry milk. Subsequently, WNV NS1 proteins were detected using 16-NS1 MAb [44] (1 μg/ml) after incubating membranes in TBST supplemented with 2.5% milk overnight at 4°C or 3 hours at room temperature. After extensive washes with TBST, membranes were incubated with HRP-conjugated goat anti-mouse IgG (1/5,000 dilution, Sigma) for one hour at room temperature. Proteins were detected using a chemiluminescent substrate (Supersignal West Femto, Thermo Scientific) and exposure to film (CL-X Posure TM Film, Thermo scientific).

### Flow cytometry analysis

To assess total levels of cellular NS1, flow cytometry was utilized. BHK21 cell lines propagating VEEV replicons that expressed WNV NS1, WNV NS1-IS (non-coding), DENV NS1, JEV NS1, SLEV NS1, or YFV NS1 were detached with HBSS containing 5 mM EDTA. Cells were washed twice with PBS on ice, fixed with 2% formaldehyde in PBS for seven minutes, and washed again with HBSS. Cells were permeablized with 0.1% (w/v) saponin and 0.1% BSA in HBSS (permeabilization buffer) and then incubated with primary MAbs (WNV E16 as an isotype control, 9NS1 as a cross-reactive anti-NS1 MAb [44], 2G6 (DENV NS1-specific) [45], 4NS1 (WNV NS1-specific) [44], 10NS1 (reacts with WNV and JEV NS1) [44], and 4E3 (YFV NS1-specific) [46] for one hour on ice. After three washes with permeabilization buffer, cells were incubated with Alexa Fluor-647 conjugated goat anti-mouse IgG (Molecular Probe) for 30 minutes and then washed three additional times. Expression levels of NS1 were determined by flow cytometry on a BD FACSArray (Becton Dickinson) and data was processed with FlowJo software (Tree Star, Inc).

### Asymmetric positive and negative strand qRT-PCR

Strand-specific real-time RT-PCR was performed as described using E gene primers [31,32]. Each transcript reaction was treated with DNAse (Ambion) thrice consecutively followed by RNA extraction with Trizol (Invitrogen) according to the manufacturer’s instruction. PCR was performed with and without reverse transcription to confirm that RNA was free of template DNA. Wild type BHK21 cells or BHK21 VEEV-WNV-NS1 cells (6 × 10^5^) were infected with WNV or ∆-NS1-WNV at an MOI of 5. Total cellular RNA was harvested at specified time points using the RNEasy mini kit (Qiagen) following the manufacturer’s instructions, and viral RNA was quantified using fluorogenic quantitative RT-PCR (qRT-PCR). Strand specificity of the qRT-PCR was confirmed using individual asymmetric primer sets and positive or negative strand RNA that was generated from *in vitro* transcription reactions after extensive DNAse treatment (data not shown).

### Virus attachment assays

BHK21 cell monolayers were detached with HBSS and 5 mM EDTA, washed once with complete DMEM, and washed three times with chilled PBS. Cells (10^6^) were aliquotted into eppendorf tubes and 10^6^ FFU of WNV or ∆-NS1-WNV was added in total volume of 100 μl. After binding for one hour, unbound virus was removed by centrifugation (200 × g) and three washes with chilled PBS. Bound virus was quantified after cell lysis and RNA purification (RNEasy Kit, Qiagen) and quantification of genomic RNA using qRT-PCR and a primer and probe set specific for positive strand specific WNV RNA as described above. Values were normalized to 18S rRNA levels to account for possible differences in cell number.

### Microscopic analysis of viral entry

BHK21 cells (6 × 10^5^) were infected with an MOI of 5 of WNV or ∆-NS1-WNV for one hour at 37°C. Unbound virus was removed after three rinses with PBS and complete DMEM was added for specified time points. Subsequently, cells were rinsed with PBS, fixed with 3% paraformaldehyde in PBS for 10 minutes, and permeabilized with PBS supplemented with 0.5% Triton-X 100. After additional washes with PBS and glycine (10 mM), cells were incubated with WNV E16 MAb or a negative control MAb (2G6, anti-DENV-2 NS1) for 20 minutes at room temperature in DMEM containing 10% BSA. After three washes with PBS and 10 mM glycine pH 7.4, cells were incubated for 15 minutes with AlexaFluor 555-conugated goat anti-mouse antibody (1/400 dilution), and nuclei were counterstained with To-PRO 3 (Molecular Probes). After several washes with PBS, cells were mounted on glass slides, and images were acquired with a laser scanning confocal microscope (Zeiss LSM 510 META) and analyzed with LSM image browser software (Zeiss).

### Radiolabeling and immunoprecipitation

To compare the initial translation rates, 6 × 105 BHK21 cells or BHK21 VEEV-WNV-NS1 cells were infected at an MOI of 5 of WNV or Δ-DNS1-WNV. At specific time points after infection, cells were starved with methionine free DMEM for 15 minutes, and then labeled for 20 minutes with 50 mCi/ml of 35S methionine and cysteine (EasyTag™ EXPRES35S Protein Labeling Mix, PerkinElmer Life Science). Labeled cells were solubilized in 800 μl of Lysis buffer (50 mM Tris-HCl, pH 8.0, 150 mM NaCl, 1% NP-40, 0.25% sodium deoxycholate, and 1 mM EDTA supplemented with a protease inhibitor cocktail (Sigma)) for 10 minutes on ice. Nuclei were removed by centrifugation (13,800 × g) for five minutes at 4°C. Subsequently, lysates were incubated with WNV E16 MAb (10 mg per sample) for three hours at 4°C. WNV E protein-MAb complexes were immunoprecipitated with protein A-Sepharose beads (Invitrogen) after an additional one-hour incubation at 4°C. Beads were washed three times with 10 mM Tris pH 7.4, 150 mM NaCl, 1% sodium deoxycholate, and 1% NP-40 and proteins were eluted by boiling samples at 95°C for five minutes in 4X SDS sample buffer supplemented with 5% (v/v) β-mercaptoethanol. Eluates were separated by 12% SDS-PAGE, gels were vacuum-dried on filter paper for one hour at 80°C, and proteins were visualized after exposure to Kodak Biomax light film.

### Expression and analysis of CMV-launched ∆-NS1-WNV-KUNV

Unique *MluI* restriction sites were generated within the NS1 sequence in the full-length WNV expression vector pKUN1 [35] at residues 4 and 298 (described in [38]) using site-directed mutagenesis. Briefly, pKUN1 was amplified with *Pfu* Ultra Hot-start polymerase (Stratagene) and primers incorporating *MluI* sequences (listed in Table 2) as follows: 92°C for 2 min then 18 cycles of 92°C for 30 s, 55°C for 1 min and 68°C for 21 min. Following restriction digestion and Sanger sequencing to verify mutations, plasmids were cleaved with *MluI* to remove the majority of NS1-coding sequence, then re-ligated and transformed into JM109 chemically-competent *E.coli* cells. Δ-NS1 plasmids were amplified and extracted using a Hi-Speed Plasmid Midiprep kit (Qiagen). A mutation (NS5 GDD to GAA) that abolishes RNA polymerase activity has been described previously [38], and was made by site directed mutagenesis.

**Table 2:** **Primers for cloning CMV-launched pKUN-**∆**-NS1**

**Primer**	**Sequence (5′ – 3′) ( *MluI *)**
NS1-Mlu1-A-forward	GCTGATACTGGATGTACGCGTGATATAAGTCGGCAAG
NS1-Mlu1-A-reverse	CTTGCCGACTTATATCACGCGTACATCCAGTATCAGC
NS1-Mlu1-B-forward	CATCGTGGACCTGCCACGCGTACCACTACAGAGAG
NS1-Mlu1-B-reverse	CTCTCTGTAGTGGTACGCGTGGCAGGTCCACGATG

Vero or BHK21-VEEV-NS1 cells were transfected with 1 μg each of pKUN1, pKUN1-NS5-GAA or pKUN1-Δ-NS1 cDNA using Lipofectamine 2000 (Life Technologies) as described by the manufacturer. Two days hours after transfection, cells were fixed in either 1:1 acetone:methanol or 4% v/v paraformeldehyde and permeabilized with 0.1% w/v Triton X-100. Cells were then incubated with MAbs to dsRNA (English & Scientific Consulting Bt. (Hungary)), NS1 and NS5 (kindly provided by R. Hall, University of Queensland) or rabbit polyclonal antisera raised against NS3 [39] or NS4A [47]. Confocal images were collected on a Zeiss confocal microscope.

### Data analysis

All data were analyzed statistically using Prism software (GraphPad4, San Diego, CA). Differences in viral infection or RNA levels were analyzed using a two tailed, unpaired *t*-test.

## Abbreviations

ER: Endoplasmic reticulum; WNV: West Nile virus; JEV: Japanese encephalitis virus; DENV: Dengue virus; YFV: Yellow fever virus; SLEV: Saint Louis encephalitis virus; NS: Non-structural; qRT-PCR: Quantitative reverse transcription polymerase chain reaction; VEEV: Venezuelan equine encephalitis virus; WT: Wild-type; KUNV: Kunjin virus; DMEM: Dulbecco’s modified Eagle medium; HRP: Horseradish peroxidase; HBSS: Hank’s balanced salt solution.

## Competing interests

The authors declare that they have no competing interests.

## Authors’ contributions

SY and RLA performed all of the experiments. SY and MSD prepared the initial draft of the manuscript, and RLA and JMM edited the manuscript into final form. MSD and JMM supported the work with research grants from the National Institutes of Health (U54-AI057160) and the National Health and Medical Research Council of Australia (No. 100461). All authors read and approved the final manuscript.
